# Chiral gliding: Right-handed navigation of filamentous cyanobacteria

**DOI:** 10.1073/pnas.2534547123

**Published:** 2026-02-26

**Authors:** Andrej Vilfan, Leila Abbaspour, Stefano Villa, Vahid Nasirimarekani

**Affiliations:** ^a^Department of Condensed Matter Physics, Jožef Stefan Institute, Ljubljana 1000, Slovenia; ^b^Laboratory for Fluid Physics, Pattern Formation and Biocomplexity (LFPB), Max Planck Institute for Dynamics and Self-Organization, Göttingen 37077, Germany

**Keywords:** gliding, chirality, cyanobacteria filament, active filaments

## Abstract

Cyanobacteria are the earliest known organisms that produced oxygen through photosynthesis, leading to the oxygen atmosphere that allowed the evolution of more complex life forms. Many species of cyanobacteria exhibit gliding motility along surfaces to navigate complex environments and adapt to fluctuating conditions. Here, we studied the gliding motility of filamentous cyanobacteria *Lyngbya lagerheimii* at the transition between different physical environments. We show that on a dry surface, a filament adopts a curved shape that turns right while gliding. When a filament switches the gliding direction, the curvature is initially preserved and a filament can turn left as long as it backtracks along a slime trace. We propose a model of chiral motility that explains the bending based on the right-handed rotation of gliding filaments and a velocity mismatch between the leading and the trailing end of the filament. The mechanism involves a unique way of transferring the structural chirality to the macroscale and also a unique physical navigation mechanism.

## Significance

Filamentous cyanobacteria use gliding motility to move along solid surfaces. They can respond to environmental stimuli by reversing the direction of motion. Our study reveals an additional mechanism by which gliding cyanobacteria can steer their movement. We show that the intrinsic chirality of a bacterium, which causes rotation about the filament axis, together with an inhomogeneity in the surrounding conditions, bends the filament such that it glides on a curved path. This chiral steering enables a unique navigation strategy that guides the filaments that encounter dry conditions back toward the favorable aqueous environments. Hence, the gliding motility exhibits a mechanism of chirality transfer from the molecular to the macroscopic scale, akin to the establishment of body asymmetry in higher organisms.

One remarkable example of biology employing intriguing physical mechanisms is the gliding of animals and organisms on a solid surface (1–3). This form of locomotion can be observed in a variety of species, ranging from bacteria, snails, and slugs to certain reptiles and fish, each employing unique biological mechanisms to traverse their habitats (4, 5). Gliding of an organism on a substrate involves complex interactions between the organism and the surface, depending on the properties of the substrate. The study of surface gliding not only sheds light on evolutionary adaptations (6, 7), but also provides insights into the fundamental physical principles governing this mode of movement.

A compelling property of the gliding motility is that it can have a chiral nature (8). The mechanism of gliding therefore belongs to the numerous independent mechanisms of chirality establishment in biology (9). These also include the handedness of snail shells (10), the body laterality in insects and invertebrates (transferred from the cellular chirality of the actin cytoskeleton) (11), of mice and fish (determined by ciliary flows) (12), as well as chirality in plant organs, such as tendrils (13) and awns (14). Nevertheless, the advantage of a defined chirality, as opposed to a random mix between left- and right-handed individuals, remains elusive (11).

Many species of bacteria use gliding motility to translocate along solid or semisolid surfaces. All forms of gliding motility are energy-dependent and many gliding bacteria rely on specialized surface proteins (1, 15) or cytoskeletal elements (16) that interact with the cell envelope or the external environment to generate movement. Unlike other motility forms such as flagellar motility [swimming (17)] and twitching [pili-based movement (18)], gliding motility is slower, typically smooth, and its mechanistic basis is not fully understood in all bacterial species (4, 19–21).

**Fig. 1.**
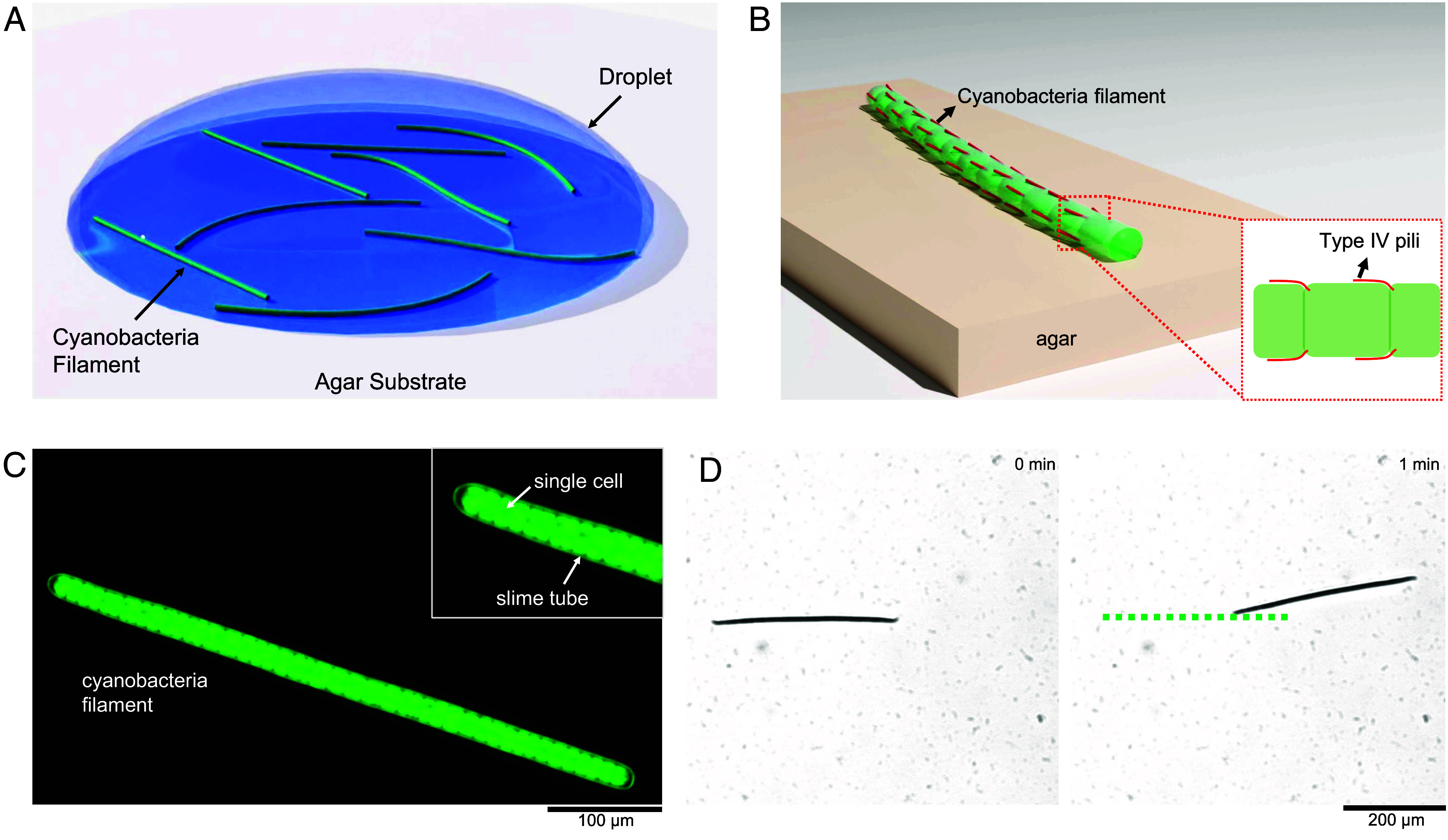
Semiflexible filamentous bacteria in transition from fully hydrated volume to a dry surface. (*A*) The droplet setup for studying the gliding pattern of the filaments in transition from the droplet to the dry surface. (*B*) Schematic of a bacterial filament on dry surface (agar substrate). (*C*) Autofluorescence microscopy of a single filamentous cyanobacteria filament (*Lyngbya lagerheimii*), highlighting single cells and presence of the slime tube in the *Inset* view. (*D*) Example of a gliding filament at the bottom of a Petri dish, in a fully hydrated volume. Although the filament remains straight, small random rotations without a determined sense of chirality are possible, especially upon direction reversal (Movie S1).

Cyanobacteria, commonly known as blue-green algae, represent a significant and ancient group within the bacterial kingdom (22). They are among the most ecologically important bacteria, contributing to photosynthesis, nitrogen fixation, and the formation of ecosystems like stromatolites (23, 24). Normally they exist in either single cell or filamentous shape. A filament consists of single cells chained up as filament with certain mechanical properties such as elasticity and flexibility (25). The mechanism of gliding motility in filamentous cyanobacteria is not yet fully understood. Explanations that have been discussed (26, 27) include the extrusion of polysaccharide slime (28) or waves passing through an array of surface fibrils (29). A mounting body of evidence, however, suggests that the gliding motility is driven by the extension, attachment, and retraction of Type IV pili (30, 31). This was shown in heterocyst-forming *Nostoc punctiforme* (32–34), as well as *Phormidium lacuna* (35) from the *Oscillatoriaceae* family. The activity of Type IV pili has been directly visualized in unicellular cyanobacteria (36). Irrespective of its role in the gliding motility, many species of filamentous cyanobacteria are known to produce and secrete extracellular polymeric substances (EPS), which can form a slimy matrix around the cells (37, 38) (Fig. 1 *B* and *C*). This EPS matrix can serve various functions, including protection from desiccation, adhesion to surfaces, and potentially facilitating gliding motility by reducing friction with the substrate and enabling the adhesion of Type IV pili.

Long filamentous bacteria are intriguing from the perspective of physics, as they are elastic (25, 39) and flexible (40, 41) filaments and self-organize to macroscopic patterns (42), such as light-induced pattern formation at illumination boundaries (43). These phenomena suggest that shape and motility change in response to forces acting on them (25, 40). The mechanical properties of these active filaments are relatively well known, but how their motility changes in different physical environments has not yet been elucidated. In other words, how physical factors affect their motility is still unclear.

We address the role of physical environment from the directionality perspective. The concept of motility is inherently linked to directionality (44), meaning that navigation is an essential part of motility allowing organisms to reach specific environments, to find nutrients (45), or to deliver cargo (46). It is known that bacteria motility on a surface can reverse its direction in response to the environmental cues such as light intensity (47), water (48) and nutrient gradients. Because the external physical conditions determine the interaction of the filament with its environment, we hypothesize that they would directly affect the motility and directionality of the gliding filaments (7), independent of the signaling pathways that process the biological triggers. In particular, we are addressing the question whether environmental cues can steer filament gliding directly in a continuous manner, in addition to the directionality reversals.

## Results and Discussion

The gliding of single filaments in fully hydrated volumes (in a Petri dish) shows that the straight shape is preserved during the gliding process, despite the flexibility of the filaments (Movie S1). The filaments show reversals of the direction and oscillation of the head and tail that allows them to change their gliding path to a certain degree (Fig. 1*D*). This points out that although the filaments are flexible, they maintain a straight shape in the fully hydrated environment. A filament that is fully surrounded by water does not experience any surface tension gradients, neither axial nor longitudinal. This suggests that the gliding forces of the filament, along the long axis, keep it straight while pushing back and forth on the substrate.

In this regard, the droplet experiment (Fig. 1*A*) allowed us to study the gliding dynamics in two different media such as the transition from a fully hydrated volume to a wet substrate in exposure to air. The activity of the filaments inside the droplet and the ones gliding out of the droplet was visualized over time using an inverted phase contrast microscope (Fig. 1 *C* and *D*). Therefore, in our experiments, the single filaments experience a transition of physical conditions and we use the results to draw a conclusion about the mechanism of navigation of these filaments on a substrate.

### Transition from Straight Shape to Right-Handed Bending and Chiral Gliding.

**Fig. 2.**
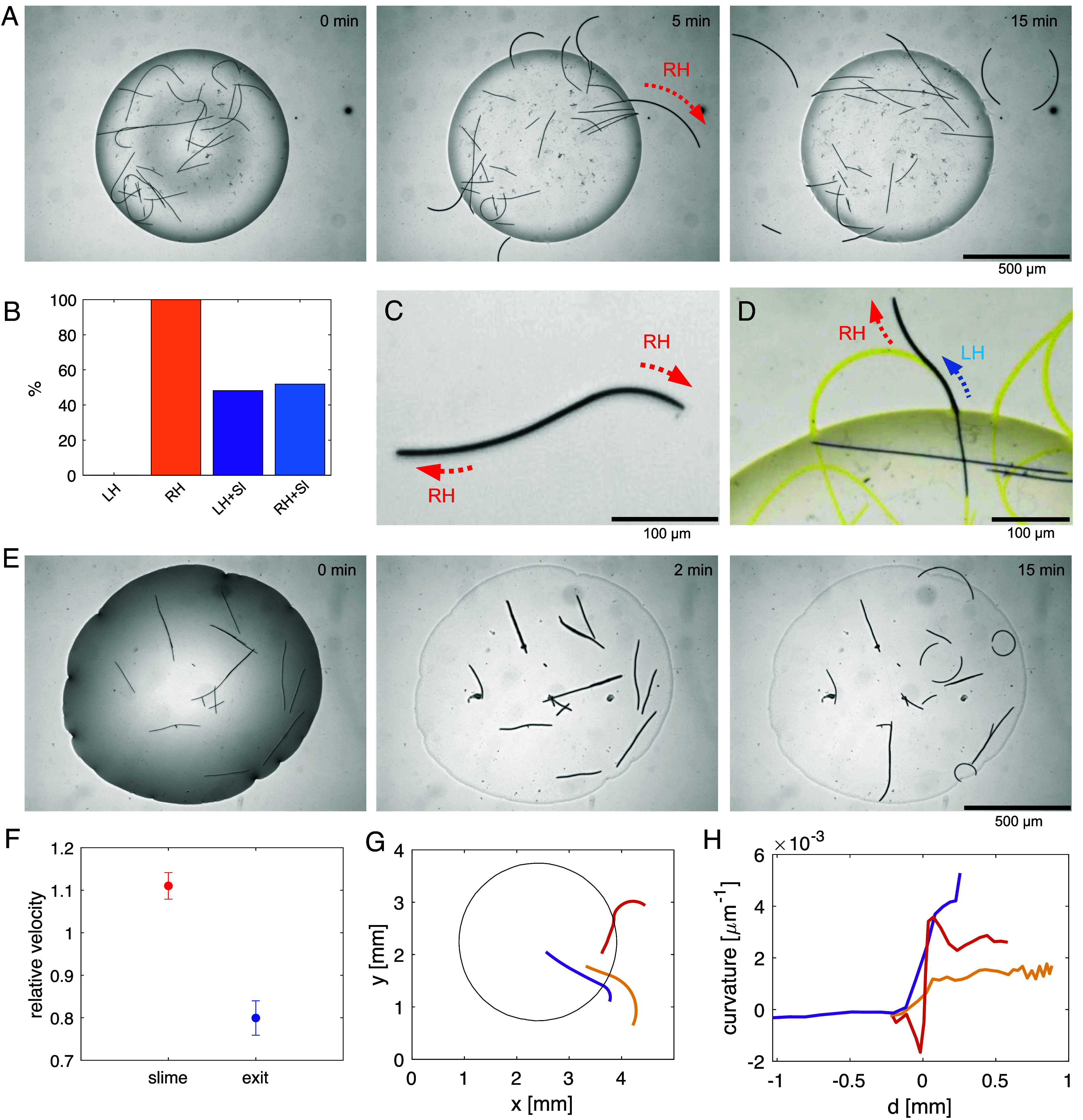
Filaments bend in a right-handed way after the transition from hydrated droplet to dry surface. (*A*) Time-lapse images of the experiment showing that the filaments leaving the droplet turn right (Movie S2). (*B*) The probability of right bending in outgoing filaments. LH and RH represent the percentage of the left- and right-handed bending filaments among the filaments that glide out of the droplet. LH+Sl and RH+Sl respectively represent the percentage of filaments that turn left while gliding back on their initial slime traces and filaments which only show right bending. (*C*) Example of a filament which turns right while gliding in one direction and then again right with the opposite end upon reversal. (*D*) A filament which initially follows a slime trace (marked in yellow) that turns it left, but then escapes it and proceeds to the right (Movie S3). (*E*) Evaporating droplet experiment in which the droplet evaporates and the filaments start to glide on the dry surface (Movie S4). (*F*) Velocity change of the filaments when they glide back on their slime traces (red) and when they exit the droplet (blue). For evaluating the relative velocities, the average velocities of the filaments back on their slime and outside the droplet have been normalized respectively over the average velocities when they are not moving on the slime and when they are inside the droplet. (*G*) Examples of filaments that bend with varying ranges of curvature in a right-handed manner as they leave the droplet. (*H*) Curvature κ of filaments from panel (*G*) as a function of the distance d from the droplet boundary (positive values refer to points outside the droplet).

The experiment with the filaments inside a droplet deposited on agar substrate shows that all filaments bend to the right upon leaving the droplet (highlighted with a red arrow in Fig. 2*A* and Movie S2). When an outgoing filament undergoes direction reversal, it initially backtracks its own trajectory, which results in left turning. In contrast, filaments inside the droplet exhibit a predominantly straight shape while gliding, occasionally showing direction changes and activity similar to that observed in the Petri-dish experiment (Fig. 1*D*).

We have tracked the filaments and mapped the cumulative gliding trajectories (Fig. 2*D* and Movie S3), which can be used as a proxy for the slime traces that a gliding filament leaves behind on the substrate (49). The filaments always bend right on “fresh” agar without previous traces (Fig. 2*B*–*D*). However, a filament that is following a slime trace in a direction that is opposite from the direction in which the trace was formed, bends left, as imposed by the trace (Fig. 2*D*). Some filaments randomly leave the trace and bend right again afterward. Likewise, filaments can switch to another trace when they encounter an intersection.

The right bending occurs when the filaments glide on the agar substrate, where they partially (with the leading tip) come into contact with air. To understand whether the bending is caused by the change of environment from a fully hydrated volume to air contact, we have conducted an experiment in which the droplet evaporates and the filaments get in contact with air. Upon complete evaporation of the buffer liquid the filaments bend right similar to the previous experiments as soon as they start gliding on the dry surface (Fig. 2*E* and Movie S4). We conclude that the trajectory curvature is directly determined by the nature of the local environment.

In addition to the directional changes, the filaments become slower upon leaving the droplet. The average velocities outside the droplet are around 20% lower than in water (Fig. 2*F*). The velocities after evaporation of a droplet are similar to those of filaments gliding out of the droplet. Following the same logic, the filaments that reenter the droplet recover their initial velocities and regain a straight shape. On the other hand, the velocities inside the droplet do not depend on the filament length. The velocity reduction is independent of the radius of curvature on agar substrate, which shows a high variability between filaments (Fig. 2 *G* and *H*). Measurement of the radius of curvature shows that the bending follows a sharp trend at the transition to the agar substrate, where the velocity reduction is pronounced (*SI Appendix*, Fig. S1). In addition, the radius of curvature could change especially if the filaments leave the slime trace they follow in a left-handed direction (a case shown in Fig. 2*D*).

The observations discussed above show, in brief, two important changes in the motility of the filaments when the physical environment changes: i) transition from a straight shape to chiral gliding on the agar substrate and ii) significant reduction of the gliding velocity. They raise two central questions: i) What is the origin of filament bending and its relation to the decreased velocity? And ii) why do the filaments always bend to the right?

### Friction Based Bending, Buckling, and Slowing Down at the Transition Interface.

We first test the hypothesis that friction in the transition from a water environment to air could slow down the gliding velocity and possibly cause bending. To examine this, we performed a 2D simulation in which a self-propelling filament encounters friction changes in order to simulate the changes that occur when the filament leaves the droplet.

In our simulation, the semiflexible filament is represented as a chain of N segments linked by harmonic springs, with its flexibility regulated by a harmonic bending potential. In the overdamped regime, the position $r_{i}$ of each segment evolves according to the following equation:

[1] $$\begin{array}{c} \gamma \left(r_{i}\right){\dot{r}}_{i}=-\nabla_{i}U_{\text{s}}-\nabla_{i}U_{\text{b}}+F_{i}^{\text{act}}-\nabla_{i}U_{\text{int}}+F_{i}^{\text{rand}}. \end{array}$$

The parameter $\gamma (r_{i})$ represents the friction coefficient of the surface and follows the sigmoidal profile:

[2] $$\begin{array}{c} \gamma \left(r_{i}\right)=\gamma_{\text{in}}+\left(\gamma_{\text{out}}-\gamma_{\text{in}}\right)\left(0.5+0.5\tanh\left(\frac{|r_{i}|-R_{\text{droplet}}}{\delta_{R}}\right)\right). \end{array}$$

**Fig. 3.**
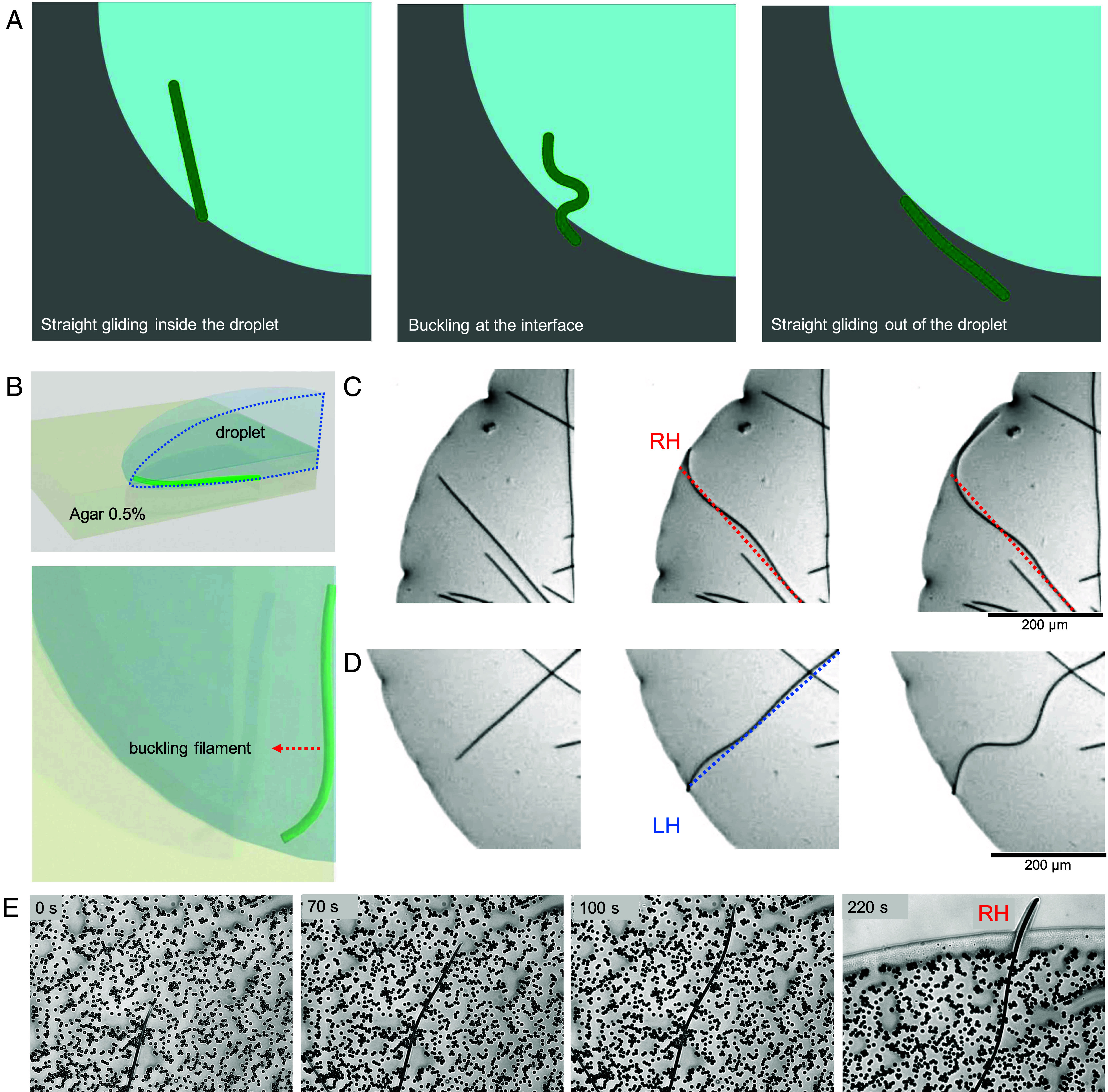
Friction and buckling effect at the transition between different environments cannot explain the chiral bending of the filament. (*A*) Snapshots from a simulation which show buckling of the filament upon a jump in the friction coefficient from a lower (blue area) to a higher value (gray area). (*B*) Schematics showing the buckling experiments at the droplet interface of the filaments entrapped inside a droplet. (*C* and *D*) Two examples of buckling filaments, highlighting the presence of both right- and left-handed buckling. (*E*) Suppression of buckling due to physical obstacles (solid particles); the filament retains its right-handed chiral gliding activity when leaving the obstacles.

Here, $\gamma_{\text{in}}$ is the friction coefficient experienced by each segment inside the droplet and $\gamma_{\text{out}}$ is the friction coefficient encountered by segments as they cross the droplet boundary and glide on agar in contact with air. It is always the case that $\gamma_{\text{out}}>\gamma_{\text{in}}$. The radial distance of segment i to the center of the droplet is $|r_{i}|$ while $R_{\text{droplet}}$ represents the radius of the droplet. The parameter $\delta_{R}$ defines the width of the transition region, ensuring a smooth change from the lower friction coefficient $\gamma_{\text{in}}$ to the higher friction coefficient $\gamma_{\text{out}}$. When $\delta_{R}=0$, the friction coefficient undergoes a sharp change at the interface. For a detailed description of the model, see *Materials and Methods*. The simulation shows that the transition of friction results in slowing down the filament and initial buckling of the filament in random orientations, either right or left (Fig. 3*A* and Movie S5). However, once the filament has left the droplet entirely and is subject to a homogeneous friction along its length, the straight shape is recovered. We conclude that friction can act as a braking mechanism at the tip of the filament which then buckles the filament as a canceling mechanism of the drag force applied at the transition. We conclude that nonuniform friction can buckle the filament, but only transiently as the filament exits the droplet.

In order to confirm that buckling in gliding filaments does not have a preferred orientation, we studied buckling of the filaments in two different experimental setups: i) at the interface of the droplet and ii) through particle obstacles (Fig. 3 *B* and *E*). For the interfacial buckling, the filaments were enclosed in a droplet on a soft agar substrate so that they could not leave the droplet (Fig. 3*B*). The results show that filaments buckle at the interface and buckling does not have a defined direction, occurring both left and right (Fig. 3 *C* and *D*). On the other hand, a filament gliding through particle obstacles on agar substrate shows that the obstacles can cause random bending of the filament (Fig. 3*E*). However, once the tip of the filament leaves the obstacles, it bends to the right (Fig. 3*E*, 220 s). Comparing these results with the previous experimental observations (Fig. 2), we conclude that the friction or physical obstacles result in buckling and slowing down the filament, but do not consistently result in right-handed gliding. The preserved right-handed gliding indicates that the bending results from a mechanism that is intrinsic to the filament and its interaction with the substrate.

### Clockwise Rotation of a Gliding Filament About Its Long Axis.

Because it is known that many species of filamentous cyanobacteria show a chiral corkscrew-like rotation during the gliding motility, we next investigate the hypothesis that the two manifestations of chiral motility are related to each other. For example, *Lyngbya aeruginosa* from the same genus as *Lyngbya lagerheimii* studied here, along with *Phormidium* and *Oscillatoria princeps* were observed to secrete slime with a helical structure that is left behind as the filament glides on a substrate (28, 50). This suggests that a filament rotates about its axis inside the slime tube. In the following, we first measure the helical rotation of filamentous *L. lagerheimii* and then devise a physical model showing how the helical rotation could lead to right-handed filament bending.

**Fig. 4.**
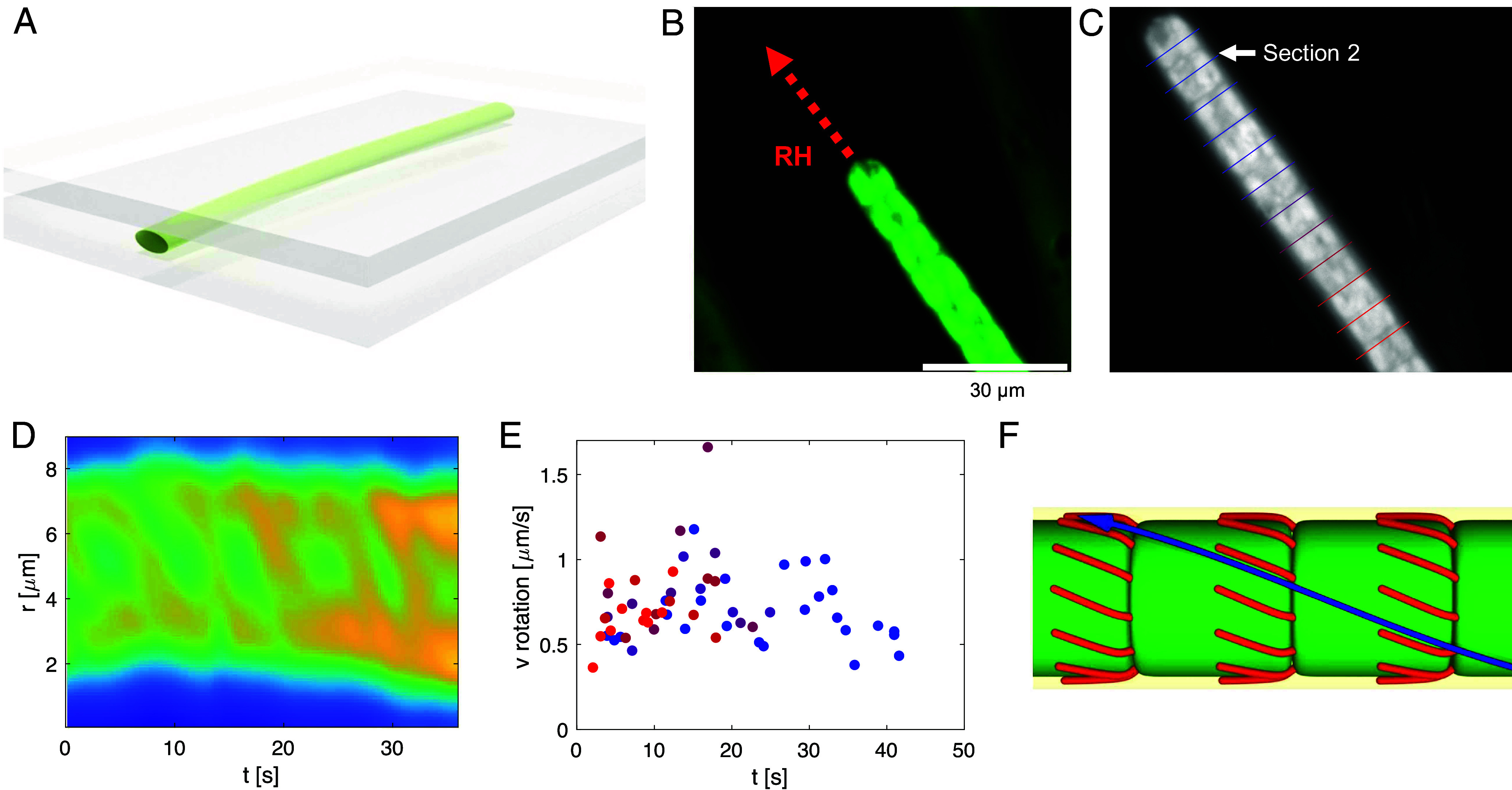
A gliding filament is rotating about its long axis. (*A*) Schematics of the Flat Cell Imaging method used to observe filament rotation. The filaments are flattened between two glass cover-slips without blocking their motility. (*B*) Linear gliding of the filament in the flattening setup. (*C*) Sectioning of the filament to quantify the rotation of the filament as it glides. (*D*) Kymograph of the section 2, showing a periodic rotation. (*E*) Surface velocity of rotation about the long axis of the filament ($v_{R}$), for all the sections shown in panel (*C*). (*F*) Proposed mechanism of rotation: Type IV pili (red) grow in chiral manner from rings near the septa between cells. As they attach to the surface and shrink, they pull the filament such that its surface motion follows a helical pattern (blue arrow).

In order to observe the rotation and quantify it, we have confined the filaments between two glass cover-slips [Flat Cell Imaging method (51), Fig. 4*A*], which allows a better visualization of the filament by its partial flattening. The gliding of a single filament along a straight trajectory demonstrates that the filaments rotate about their long axes inside the slime tube while maintaining a straight shape (Fig. 4*B* and Movie S6). Rotation is clockwise in the direction of gliding, making the filaments right-handed. Moreover, the reversal of the gliding direction preserves the chirality—rotation after reversal is again clockwise with respect to the reversed gliding direction, consistent with observations in *Fluctiforma draycotensis* (52). The reversal is analogous to a screw-nut mechanism, in which chirality is always maintained with respect to the gliding direction (*SI Appendix*, Fig. S3).

By real-time object registration of the filament shown in Fig. 4*B*, we quantified the rotation of the filament in different sections along the filament (Fig. 4*C*). The kymograph of an example section shows periodic rotation of the filament about its long axis (Fig. 4*D*). The rotational velocities have an average of $v_{\text{R}}=0.8\;\mu \text{m}/\text{s}$ in all sections along the filament, in the case of a gliding velocity measured around $v_{\text{T}}=1.5\;\mu \text{m}/\text{s}$. (Fig. 4*E*). The ratio between these velocities determines a helix angle $\beta =\;\arctan(v_{\text{T}}/v_{\text{R}})$ of around 60 degrees, similar to reported results from other species of rotating bacteria (53, 54).

Bacterial rotation can be seen in various species with or without flagella (55, 56), both in right- and left-handed sense (28). Here, the coincidence of the clockwise rotation about the long axis of the filament with right-turning gliding suggests a relationship between these two observations. We assume that the arrangement of type IV pili that is skewed clockwise generates upon surface contact a force inclined at an angle relative to the filament axis (Fig. 4*F*). Because the slime envelope largely suppresses lateral translation of the filament, the sideways force leads to axial rotation. Together with translation, the filament moves in a corkscrew-like motion, representing a new form of motility that is distinct from twitching. Nevertheless, the rotation alone is not sufficient to bend the filament to the right (Fig. 4*C*). Rather, this bending results from external physical parameters.

### Axial Torque and Clockwise Rotation Result in Bending of the Filament.

A closer look at the filament on agar substrate shows the presence of an asymmetric surface tension acting on the filament (Fig. 1 *B* and *C*). The slime tube exposed to air has a lower surface tension than the slime–agar contact point (7, 57). Therefore, we hypothesize that the agar exerts a drag on the rotation of the filament, which then causes a clockwise axial torque. Given the flexibility of the filament, the radial torque then results in right-handed bending of the filament.

**Fig. 5.**
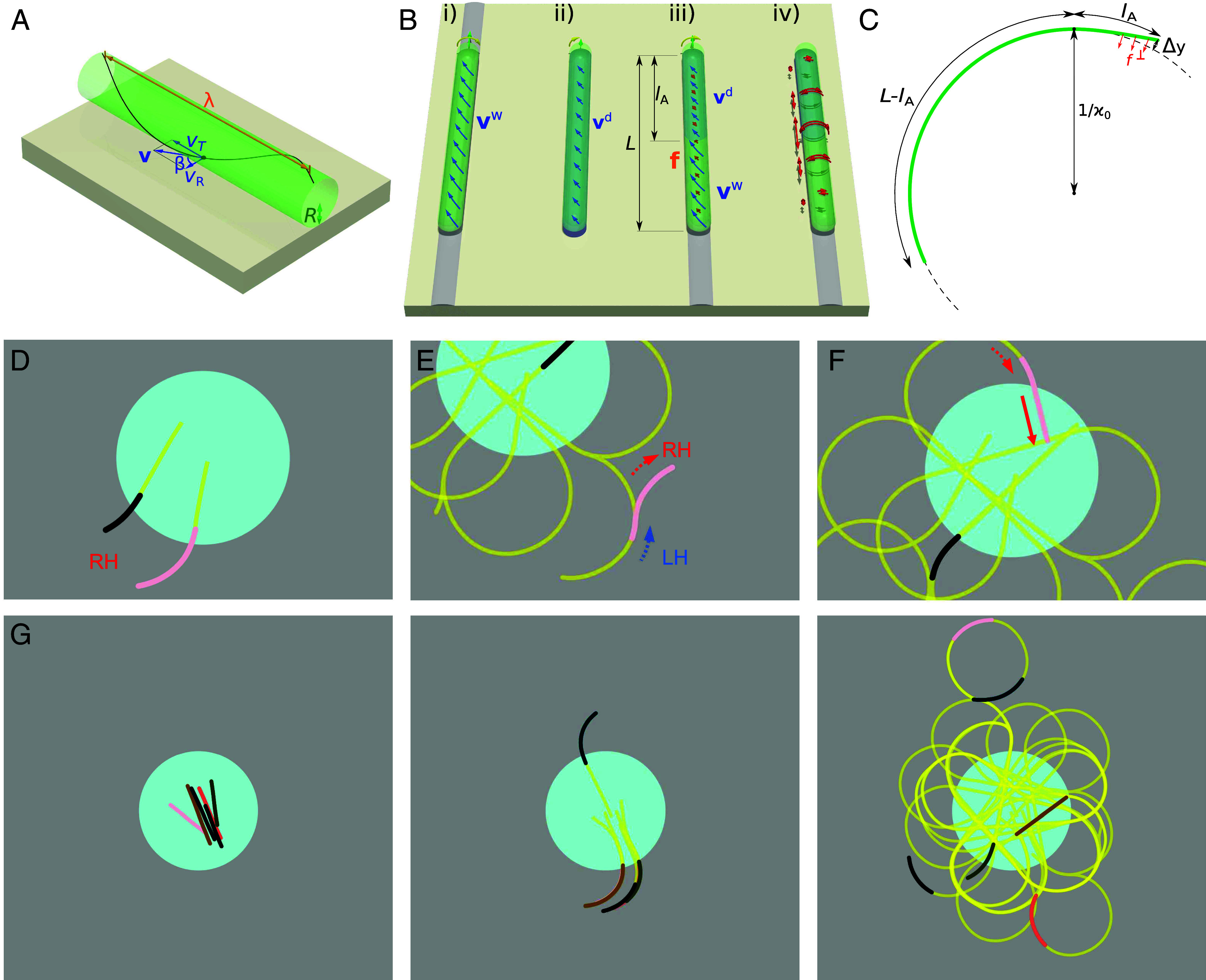
Model based on clockwise helical rotation and a velocity mismatch between the leading end and the rest of the filament explains the right-handed bending of the filament. (*A*) Helical gliding of a filament. Each point on the surface of the filament moves with the translational velocity $v_{\text{T}}$ and rotational (lateral) velocity $v_{\text{R}}$. Its trajectory follows a helix with pitch λ, radius R, and helix angle β. (*B*) Velocity mismatch mechanism: *i*) Filament in water or following a slime trace glides in a helical way with surface velocity $v^{\text{w}}$ (blue arrows). *ii*) On a dry surface, the velocity is reduced to $v^{\text{d}}$, while the pitch of the helix remains unaffected. *iii*) A filament gliding along a dry surface would have an unloaded sliding velocity $v^{\text{d}}$ in the leading part and $v^{\text{w}}$ in the trailing part (illustrated with a sharp transition). As a result, the filament glides with an intermediate velocity v and the mismatch causes a friction force f (orange). *iv*) As a result, the filament is subject to compressive force and torsion (red arrows). (*C*) Schematic representation of a filament gliding on a circular trajectory with a radius of curvature $1/\kappa_{0}$. The front part with length $l_{A}$, effectively moving along a dry surface, is subject to a lateral force $f^{⊥}$. (*D*) Simulation results showing two filaments bending right as they leave the droplet (blue). The traces of the filaments are marked in yellow (Movie S7). (*E*) An example of a filament backtracking on its slime trace and bending right on fresh agar after leaving the trace. (*F*) Recovery of the straight shape as the filament reenters the droplet. (*G*) Time lapse of the simulation of a droplet with 6 filaments (Movie S8).

To explain the transfer of chirality from the helical gliding motion of a filament to the right-curved path, we propose a model that is based on two key observations. First, the helicity of the path itself (Fig. 4) and, second, the reduction of velocity when the filament leaves the aqueous environment (Fig. 2). We start with a straight filament of length L, with a leading segment of length $l_{A}$ exposed to a dry surface. The unloaded local sliding velocity of the dry part is $v^{\text{d}}$ and of the wet part $v^{\text{w}}$. Both velocities have a direction that is inclined by an angle β= arctan(λ/2πR) from the normal to the filament (Fig. 5*A*). Here, R denotes its radius and λ the pitch of the helix. Following earlier models (40), we propose that a local deviation from the active velocity leads to a linear drag with the drag coefficient Γ. If a part of the filament glides with a different velocity v, it is subject to a local force density (force per unit length) $f=\Gamma (v^{\text{w}}-v)$ in the wet part and $f=\Gamma (v^{\text{d}}-v)$ in the dry one (Fig. 5*B*). The force balance on the filament determines the gliding velocity as $v=\left(l_{A}/L\right)v^{\text{d}}+\left(1-l_{A}/L\right)v^{\text{w}}$. In other words, the filament is gliding with a velocity that is the weighted average of the characteristic gliding velocities of its two parts. Because all external forces act on the filament horizontally at the same distance from its axis, the force balance automatically ensures that the axial torque balance is also satisfied in a nearly straight filament.

Based on the above considerations, we formulate a model for the curved gliding motility of a filament on a dry surface (Fig. 5*C*). We assume that the leading end is relatively short $l_{A}\ll L$ and does not significantly affect the velocity and rotation rate. The lead end of the filament is now subject to a mismatch $\Delta v_{\text{T}}=v_{\text{T}}^{\text{w}}-v_{\text{T}}^{\text{d}}$ between its imposed translational velocity $v_{\text{T}}^{\text{w}}$ and its own characteristic translational velocity $v_{\text{T}}^{\text{d}}$. The corresponding mismatch in rotational velocity components is $\Delta v_{\text{T}}\cot\beta$. Reduced by the actual transverse velocity $v^{⊥}$, it determines the lateral force density as

[3] $$\begin{array}{c} f^{⊥}=\Gamma \left(\Delta v_{\text{T}}\cot\beta -v^{⊥}\right). \end{array}$$

We now use the model of a flexible filament with a bending modulus EI, such that the local bending moment is M=EIκ, where κ is the curvature. We solve the beam equations to determine the steady state in which the filament follows a circular trajectory with radius $\rho =1/\kappa_{0}$. In the leading part of the filament, before constant curvature is reached, the lateral velocity is

[4] $$\begin{array}{c} v^{⊥}=V\int_{L-l_{A}}^{x}dx^{\prime }\left(\kappa_{0}-\kappa \left(x\right)\right). \end{array}$$

The elastic beam equation, on the other hand, reads

[5] $$\begin{array}{c} EI\frac{d^{2}}{dx^{2}}\kappa \left(x\right)=f^{⊥}. \end{array}$$

In the following, we assume that a segment of length $l_{A}$ at the lead end has a slower propulsion velocity and at the same time lacks the “pinning” force that keeps the filament on its own track. The rest of the filament is subject to another force that keeps it on its own track. The exact properties of the pinning force are not important for the shape of the filament and we therefore do not formulate them specifically. The beam equation for the leading part of the filament that is not pinned is obtained by combining Eqs. **3** and **5** while neglecting the effect of $v^{⊥}$:

[6] $$\begin{array}{c} \kappa^{\prime \prime }\left(x\right)=\frac{\Gamma \Delta v_{\text{T}}\cot\beta }{\mathrm{EI}} \end{array}$$

with the boundary conditions κ(L)=0 (torque-free end) and $\kappa^{\prime }\left(L\right)=0$ (force-free end). It is solved by $\kappa \left(x\right)=\frac{1}{2}\left(\Gamma \Delta v_{\text{T}}\cot\beta /EI\right){\left(L-x\right)}^{2}$ for $x>L-l_{A}$ and $\kappa \left(x\right)=\kappa_{0}$ otherwise with

[7] $$\begin{array}{c} \kappa_{0}=\frac{1}{2}\left(\Gamma \Delta v_{\text{T}}\cot\beta /EI\right)l_{A}^{2}. \end{array}$$

We can use experimentally available parameter values to estimate the length $l_{A}$ of the segment with reduced propulsion. The bending stiffness has been measured as $EI\approx 5\times 10^{-17}\;\;{\text{Nm}}^{2}$ (25). For the drag density we use a typical value of $\Gamma =1,000\;\;{\text{Nsm}}^{-2}$ (40) and assume it to be constant along the filament. For the angle we use $\cot\beta =v_{\text{R}}/v_{\text{T}}=0.53$ and for the velocity difference $\Delta v_{\text{T}}=1\;\mu \text{m}/\text{s}$. From Eq. **7**, we obtain a curvature of $\kappa_{0}=10^{4}\;{\text{m}}^{-1}$ and a length $l_{A}=43\;\mu \text{m}$. The distance of the leading tip from the extrapolated circular arc can be obtained by twofold integration

[8] $$\begin{array}{c} \Delta y=\int_{L-l_{A}}^{L}dx\int_{L-l_{A}}^{x}dx^{\prime }\left(\kappa_{0}-\kappa \left(x^{\prime }\right)\right)=\kappa_{0}l_{A}^{2}/4\approx 5\;\mu \text{m}, \end{array}$$

only somewhat larger than the thickness of the filament.

We next expanded the stochastic simulation to incorporate the above properties. We introduced a field c that describes the slime concentration outside the droplet. It starts with c=0 and then increases with time when a field is covered by a segment of a filament, until it saturates at a finite concentration. A slime concentration gradient exerts a force on the filament, leading to a tendency to follow existing slime traces. In addition, segments gliding over a surface with c<1 are subject to a perpendicular force that turns them right, representing the effect of the force $f^{⊥}$. The simulation is described in *Materials and Methods*.

The simulation reproduces well all major experimental observations. Filaments glide straight inside the droplet, but start turning right on circular trajectories as soon as they leave the droplet (Fig. 5 *D* and *G* and Movies S7 and S8). Upon reversal, the filaments initially backtrack their slime traces, but sometimes leave them and proceed turning right (Fig. 5*E*). When they reenter the droplet, they recover the straight shape (Fig. 5*F*).

We conclude that the velocity mismatch between the leading end and the rest of the filament provides an explanation for the right-handed bending of a gliding filament. Although axial torque is at its core, the bending mechanism is distinct from the classical twist-bend coupling models (58), because the bending moment results from the forces acting between the filament and the substrate. We were further able to verify the viability of the proposed mechanism with a quantitative model. Qualitatively, the picture is also consistent with the dynamics of a filament as it reverses the directionality or encounters an existing slime trace on the surface. The nonuniform gliding velocity along the length of a filament can therefore transfer the chirality of the right-handed helical motion of the filament, which occurs at a scale of ∼10 μm, to the circular trajectories of filaments at a scale of ∼100 μm or larger.

## Conclusions

Our experimental study revealed an intricate physical mechanism that utilizes the helical rotation of gliding cyanobacteria to control the steering of their trajectories. This chiral steering represents a unique navigation strategy. In favorable water environments, a filament glides along a straight path. When leaving the water droplet, a trajectory that is curved to the right helps steer the filament back toward the water. The ability to preserve the sense of rotation of the trajectory is even more surprising in view of the fact that the filament itself is symmetric and does not have a preferred gliding direction. It achieves this by leaving behind a slime trace that allows the filament to distinguish between forward motion, where it always steers right, and backtracking, where it can also turn left in the opposite direction.

The sense of helical rotation of gliding cyanobacteria is directly linked to the helical structure of the carbohydrate fibrils on their surface, which in turn are aligned by the structure of underlying glycoproteins (59). The mechanism we study here further transfers this chirality to the trajectory of a filament. The mechanism of chirality transfer from the molecular to cellular scale and eventually to the scale of the trajectory represents another example of the diverse and evolutionary convergent mechanisms of chirality establishment in biology (9). Whereas the question of whether a defined chirality is advantageous over a random mix of left- and right-handed individuals remains open in most species, the multicellularity of filamentous cyanobacteria points to a clear advantage. The model we propose requires that all cells forming a filament share identical chirality. Beyond the scope of the single filament behavior we studied here, a defined handedness of the trajectory will also have a major contribution to the ability of filaments to form collective colonies such as toruses (40) or reticulate patterns (60), which can aid survival under challenging environmental conditions. The role of chirality in the collective behavior of filamentous cyanobacteria therefore poses an intriguing question for future research.

## Materials and Methods

### Material.

Xanthan gum from *Xanthomonas campestris* and agarose were purchased from Sigma Aldrich. Glass slides and 1 micron beads were purchased from Bangs Laboratories, Inc. Microscope coverslips (purchased from Paul Marienfeld GmbH & Co. KG, 24×60 mm with thickness of 0.170 mm ± 0.005 mm) were used in the Flat Cell Imaging method (51).

### Bacteria Culture.

The species of cyanobacteria, *L. lagerheimii*, was obtained from The Culture Collection of Algae at Göttingen University and seeded in T175 culture flask with standard BG-11 (Gibco™, purchased from ThermoFisher) nutrition solution. The culture medium was exchanged for fresh BG-11 every four weeks. Cultures were kept in an incubator with an automated 12 h day (30 % light intensity, corresponding to around 20 $\mu \text{E}\;{\text{m}}^{-2}{\text{s}}^{-1}$, 18 °C) and 12 h night (0 % light intensity, 14 °C) cycle, with a continuous 2 h transition.

### Droplet Experiments.

#### Xanthan droplet.

A xanthan solution was prepared by dissolving the xanthan powder in BG-11 buffer, with 0.7% w/w concentration. Bacteria filaments were then added to the xanthan mixture and vortexed to achieve a relatively homogeneous mixture of the filaments inside the gel. Droplets of the gel and bacteria mixture, in the range of few microliters, were pipetted on the agar substrate inside a Petri dish. The agar dish was prepared as the standard method in the BG-11 media as well. Media were used to provide nutrients to the bacteria, in order to keep them active over days.

#### Evaporating droplet.

In the case of the evaporating droplet, the bacteria filaments in the media were pipetted in microliter volume droplets on the agar substrate. The droplet volume then was evaporated in few minutes and bacteria filaments started to glide on the substrate after total desiccation of the droplet volume.

### Microscopy.

#### Flattened bacteria microscopy.

Image acquisition was performed using an inverted fluorescence microscope Olympus IX-71 with a 60× oil objective (Olympus, Japan), with various binning and window size depending on the experiment. For excitation, a Lumen 200 metal arc lamp (Prior Scientific Instruments, U.S.A.) was applied. The images were recorded with a CCD camera (CoolSnap HQ2, Photometrics). The videos were acquired at different frame rates. For fast acquisition, the minimum possible interval was 300 ms.

#### Phase contrast microscopy.

Olympus CKX41 microscope with a mounted Axiocam MRm camera and a phase contrast ring was used for life cycle studies of filament gliding in droplet experiments.

### Image Processing.

The slime tracks are reconstructed and visualized as follows. If $I_{n}$ is the grayscale intensity of a pixel inframe n, with values $I_{n}\in \left[0,1\right]$, the color of the same pixel in the processed video is determined as

[9] $$\begin{array}{c} C_{n}=\left(R_{n},G_{n},B_{n}\right)=\left(I_{n},I_{n},\min_{m=1,\ldots ,n}I_{m}\right). \end{array}$$

Here, the variables $R_{n}$, $G_{n}$, and $B_{n}$ represent the intensities of the red, green, and blue channel, each again on the interval [0,1]. The conversion ensures that a pixel that is brighter than its cumulative minimum intensity in the past is colored such that the white color is turned into yellow.

### Simulation.

#### 2D friction model.

In the overdamped regime, the position of each segment, $r_{i}$, evolves according to the following equation of motion:

[10] $$\begin{array}{cc} \frac{dr_{i}}{\mathrm{dt}} & =\frac{1}{\gamma (r_{i})}f_{i}\left(t\right)+\sqrt{2D}\;\eta_{i}\left(t\right), \end{array}$$

[11] $$\begin{array}{cc} f_{i}\left(t\right) & =-\nabla_{i}U_{s}-\nabla_{i}U_{b}+F_{i}^{\mathrm{act}}-\nabla_{i}U_{\mathrm{int}}. \end{array}$$

Here, $\gamma (r_{i})$ denotes a position-dependent friction coefficient (see Eq. **2** in the main text), $D=k_{B}T/\gamma$ is the associated translational diffusivity and $\eta_{i}\left(t\right)$ a vector of Gaussian white noise processes with zero mean and unit variance. The total force $f_{i}\left(t\right)$ consists of multiple contributions: The stretching potential, $U_{\text{s}}$, results from the harmonic springs linking adjacent segments and is expressed as

[12] $$\begin{array}{c} U_{\text{s}}=\frac{K_{\text{s}}}{2}\sum_{j=2}^{N}{\left(r_{j,j-1}-\sigma \right)}^{2}, \end{array}$$

where $r_{j,j-1}=|r_{j,j-1}\left|=\right|r_{j}-r_{j-1}|$ is the distance between the j-th and (j−1)-th segments. The parameter σ represents the spacing between unstrained segments and is set to be 1. $K_{\text{s}}$ is the stretching constant, which is chosen sufficiently high to ensure that the filament is not stretchable. The harmonic bending potential, $U_{\text{b}}$, determines the bending stiffness and is characterized by the stiffness parameter $K_{\text{b}}\equiv EI/\sigma$. It is given by

[13] $$\begin{array}{c} U_{\text{b}}=\frac{K_{\text{b}}}{2}\sum_{j=2}^{N-1}{\left(\theta_{j}-\pi \right)}^{2}, \end{array}$$

where π is the equilibrium angle of between adjacent pairs of monomers, corresponding to a straight chain, and $\theta_{j}$ denotes the angle formed by three consecutive segments (j−1,j,j+1):

[14] $$\begin{array}{c} \theta =\;\arccos\left(\frac{r_{j-1,j}\;\cdot \;r_{j+1,j}}{r_{j-1,j}r_{j+1,j}}\right). \end{array}$$

Self-propulsion is generated by the active force $F_{i}^{\text{act}}$, defined as

[15] $$\begin{array}{c} F_{i}^{\text{act}}=sf_{\text{act}}\;{\hat{t}}_{i}, \end{array}$$

where $f_{\text{act}}$ sets the force magnitude and s=±1 the direction of gliding (s≡+1 in the absence of reversals). The tangent vector at the i-th segment is approximated as

[16] $$\begin{array}{c} {\hat{t}}_{i}=\frac{1}{2}\left(\frac{r_{i+1,i}}{r_{i+1,i}}+\frac{r_{i,i-1}}{r_{i,i-1}}\right). \end{array}$$

The excluded volume interaction is represented by a soft isotropic repulsion with the potential

[17] $$\begin{array}{c} U_{\text{int}}\left(r_{i,j}\right)=\left\{\begin{array}{cc} U_{0}{\left(r_{i,j}/r_{c}-1\right)}^{2} & \text{if}r_{i,j}\leq r_{c},\\ 0 & \text{otherwise}. \end{array}\right. \end{array}$$

Here, $r_{i,j}$ denotes the distance between a pair of segments and the parameter $r_{c}=2^{1/6}\sigma$ sets the interaction range.

#### Simulation of filament motion influenced by slime concentration.

In the second scenario, we expand the model to take into account two key observations, namely the tendency of filaments to follow existing slime traces and their tendency to turn right in the absence of water or a slime trace. We model the attraction to a slime trace with an additional force $\psi \gamma \nabla c(r_{i},t)$, where c(r,t)∈[0,1] is the slime concentration on the surface outside the droplet. The lateral force that results from the velocity mismatch while gliding on dry surface (c<1) is determined by Eq. **3** in the theoretical model. We describe it as $s\varphi \gamma {\hat{n}}_{i}\left[1-c(r_{i}\left(t\right),t)\right]$, where ${\hat{n}}_{i}={\hat{t}}_{i}\times {\hat{e}}_{z}$ is a rightward pointing normal to the filament. The modified equation of motion, Eq. **10**, then reads

[18] $$\begin{array}{cc} \frac{dr_{i}}{\mathrm{dt}} & =\frac{1}{\gamma }f_{i}\left(t\right)+\sqrt{2D}\;\eta_{i}\left(t\right)+\psi \nabla c\left(r_{i},t\right)+s\varphi {\hat{n}}_{i}\left[1-c(r_{i}\left(t\right),t)\right]. \end{array}$$

The slime concentration c(r,t) is defined on a discrete lattice outside the droplet. The concentration at site (x,y) evolves according to

[19] $$\begin{array}{c} \frac{dc_{x,y}}{\mathrm{dt}}=\alpha (1-c_{x,y})n_{x,y}, \end{array}$$

where α is the slime production rate and $n_{x,y}$ is the total number of segments over the lattice site (x,y). This equation describes slime secretion by the filaments once they exit the droplet, with the term (1−c) ensuring that the slime concentration saturates at a maximum value of 1. The simulation is initiated with c=0 across the whole field.

We model random reversal events—where the gliding direction of a filament instantaneously reverses—as a telegraph process with reversal rate $\lambda_{r}$ (see ref. 41 for more details). The polarity (head–tail) state $s_{m}$ of filament m follows a stochastic process characterized by instantaneous flips between +1 and −1, both with rate $\lambda_{r}$. The waiting times τ between consecutive reversal events are therefore exponentially distributed, with a probability density function

[20] $$\begin{array}{c} P_{w}\left(\tau \right)=\lambda_{r}e^{-\lambda_{r}\tau }. \end{array}$$

Collectively, these variables and terms capture the complex interplay of deterministic forces, random fluctuations, directional biases, and slime concentration governing the segment dynamics.

All simulations were conducted in a two-dimensional periodic domain of size 1,000σ and in a droplet with radius $R_{\text{droplet}}=100\;\sigma$. To capture the polydispersity in filament lengths observed experimentally, filament lengths in the simulations were sampled from a Poisson distribution with a fixed mean of 100σ. With the total number of filaments set to $N_{f}=2$ to 6, each simulation system typically consisted of approximately 200 to 800 monomers.

We adopt σ and $\sigma \gamma /f_{\text{act}}$ as the fundamental units of length and time, respectively. In these units, the dimensionless form of Eq. **18** depends on five key nondimensional parameters:

$$\begin{array}{cccccccc} {\tilde{K}}_{\text{s}} & =\frac{K_{\text{s}}\sigma }{f_{\text{act}}}, & {\tilde{K}}_{\text{b}} & =\frac{K_{\text{b}}}{\sigma f_{\text{act}}}, & {\tilde{U}}_{0} & =\frac{U_{0}}{\sigma f_{\text{act}}}, & \\ \tilde{D} & =\frac{\gamma }{\sigma f_{\text{act}}}D, & \tilde{\alpha } & =\alpha \frac{\sigma \gamma }{f_{\text{act}}},\\ \tilde{\varphi } & =\frac{\varphi \gamma }{f_{\text{act}}}, & \tilde{\psi } & =\frac{\psi \gamma }{\sigma f_{\text{act}}}, & {\tilde{\gamma }}_{\text{in}} & =\frac{\gamma_{\text{in}}}{\gamma }, & {\tilde{\gamma }}_{\text{out}} & =\frac{\gamma_{\text{out}}}{\gamma }. \end{array}$$

The specific values used for these parameters are summarized in Table 1.

**Table 1.** Simulation parameters corresponding to the data shown in the main text (unless specified otherwise)

Parameter	${\tilde{K}}_{\text{s}}$	${\tilde{K}}_{\text{b}}$	${\tilde{U}}_{0}$	$\tilde{D}$	$\tilde{\alpha }$	$\tilde{\varphi }$	$\tilde{\psi }$	$\lambda_{r}$	${\tilde{\gamma }}_{\text{in}}$	${\tilde{\gamma }}_{\text{out}}$
Value	1,000	10	10	$5\cdot 10^{-6}$	1	1	2 to 5	0.0005	0.5	1

The equations of motion were integrated using a time step of $dt=10^{-3}$, typically over $10^{7}$ steps. The relatively large value of ${\tilde{K}}_{\text{s}}=500$, listed in Table 1, was chosen to effectively suppress filament stretching.

### Analysis of the Trajectories.

In order to recover the speed and the direction of the bacteria inside and outside the droplet, a dedicated tracking algorithm has been written in MATLAB (MathWorks Inc.). The time lapse videos were first prepared with a background subtraction, evaluated as a time average of the frames after a Gaussian smoothing. Subsequently, the bacteria have been isolated via a proper thresholding binarization frame by frame. The displacement of each leading extremum of the bacteria has then been identified through image subtraction of consecutive binarized frames. An opening operation has been put in place to remove spurious signals (small lateral sliding of the bacteria, debris,...). Finally, isolated leading extrema from different frames have been automatically linked in trajectories using a distance and direction criterion. Eventual errors in linking the trajectories across time have been corrected via a visual inspection by the operator. For evaluating the instantaneous curvature of the bacteria, at each time t of each trajectory, the radius of the circle passing from the leading extremum coordinates at time t−1, t, and t+1 has been evaluated. The curvature is then defined as the reciprocal of the radius and the sign is assigned depending on the traveling direction along the circle. For evaluating the overall curvature as a function of the distance d from the droplet boundary, all the curvatures from all the trajectories have been considered (N=2 independent experiments with tens of trajectories each). The curvatures have then been binned depending on d (negative values when inside the drop). For each bin, the median value of the absolute values of the curvature has been calculated and reported in the figure.

The statistics on left/right turning outside the droplet (Fig. 2*B*) were obtained as follows. For each trajectory entirely or partially outside the droplet for at least 5 consecutive frames, the median curvature ${\overset{¯}{c}}_{O}$ outside the droplet was computed. Then, the first condition (left-handed, no slime) label was assigned to trajectories with ${\overset{¯}{c}}_{O}>0$ and not moving on the slime. The same was done for the second condition (right-handed, no slime), but now with ${\overset{¯}{c}}_{O}<0$. The third (fourth) condition was then defined for ${\overset{¯}{c}}_{O}>0$ (<0) and with more than 50% of the trajectory lying on the slime. In this analysis, 41 different trajectories have been considered from two independent experiments.

The average relative velocity in Fig. 2*F* has been obtained by selecting only the trajectories crossing the droplet perimeter. For each trajectory the frames have been identified when the bacterium was inside or outside the droplet, and when it was gliding on the slime or not. The median value of the velocity was then computed for the 4 conditions: outside without slime (${\overset{¯}{v}}_{O}$), outside with slime (${\overset{¯}{v}}_{\mathrm{OS}}$), inside without slime (${\overset{¯}{v}}_{I}$), and inside with slime (${\overset{¯}{v}}_{\mathrm{IS}}$). The relative velocity with/without slime was defined as $\left({\overset{¯}{v}}_{\mathrm{OS}}/{\overset{¯}{v}}_{O}+{\overset{¯}{v}}_{\mathrm{IS}}/{\overset{¯}{v}}_{I}\right)/2$ and the relative velocity outside/inside the droplet as $\left({\overset{¯}{v}}_{\mathrm{OS}}/{\overset{¯}{v}}_{\mathrm{IS}}+{\overset{¯}{v}}_{O}/{\overset{¯}{v}}_{I}\right)/2$. These measures were chosen to separate the effect of slime from that of being inside or outside the droplet. The mean values of the relative velocities as defined above, averaged over all trajectories, are shown in Fig. 2*F*, along with their SD.

### Rotation of the Flattened Bacteria.

The rotational dynamics of the flattened bacteria is accessible by observing the movement of the dark spots in the direction orthogonal to the filament axis. To quantify it, the time lapse videos were first registered using a space correlation method to remove the bacterium overall displacement. From that, the gliding velocity $v_{\text{T}}=1.5\mu \text{m}/\text{s}$ has been obtained. On the registered video, equally spaced lines orthogonal to the bacteria filament direction were then selected (Fig. 4*C*) and kymographs were extracted for each of them (Fig. 4*D*). The filament’s rotational velocity was then identified with the dark spots’ velocity assuming a flat filament. This was confirmed by the slope of the stripes in the kymograph, as no signs of acceleration were observed when the dark spots moved from the side to the center of the filament.

## Supplementary Material

Appendix 01 (PDF)

Movie S1. Nonpolar gliding of a single filament on the bottom of a Petri dish.

Movie S2. Right-handed turning of the filaments upon leaving the droplet.

Movie S3. Reconstruction of the slime traces.

Movie S4. Evaporating droplet experiment showing chiral gliding of the filaments.

Movie S5. Buckling of the filaments at the interface of the droplet on soft agar.

Movie S6. Clockwise rotation of the filament about its long axis.

Movie S7. Simulation results of chiral gliding filaments (2 filaments).

Movie S8. Simulation results of chiral gliding filaments (6 filaments).

Movie S9. Chiral gliding and recirculation of filaments within the droplet in a densely populated droplet.

## Data Availability

All study data are included in the article and/or supporting information.
