# Supplementary material for: Chiral gliding: Right-handed navigation of filamentous cyanobacteria
Source: Proc Natl Acad Sci U S A. 2026 Feb 26;123(9):e2534547123. doi: 10.1073/pnas.2534547123 (PMC12956854; doi:10.1073/pnas.2534547123)
Supplement: Supplementary file 1 — Appendix 01 (PDF) [file pnas.2534547123.sapp.pdf]

PNAS

Supporting Information for

Chiral gliding: right-handed navigation of filamentous cyanobacteria

Andrej Vilfan, Leila Abbaspour, Stefano Villa and Vahid Nasirimarekani

Vahid Nasirimarekani
E-mail: vahid.nasirimarekani@ds.mpg.de

This PDF file includes:

Supporting text
Figs. S1 to S4
Legends for Movies S1 to S9

Other supporting materials for this manuscript include the following:

Movies S1 to S9

Supporting Information Text

Velocity and radius of curvature for right-hand bending filaments. We measured the radius of curvature for bending filaments and then took the overall average of the curvature radii for a given distance from the droplet boundary. The curvature increases sharply at the interface (Fig. S1a).

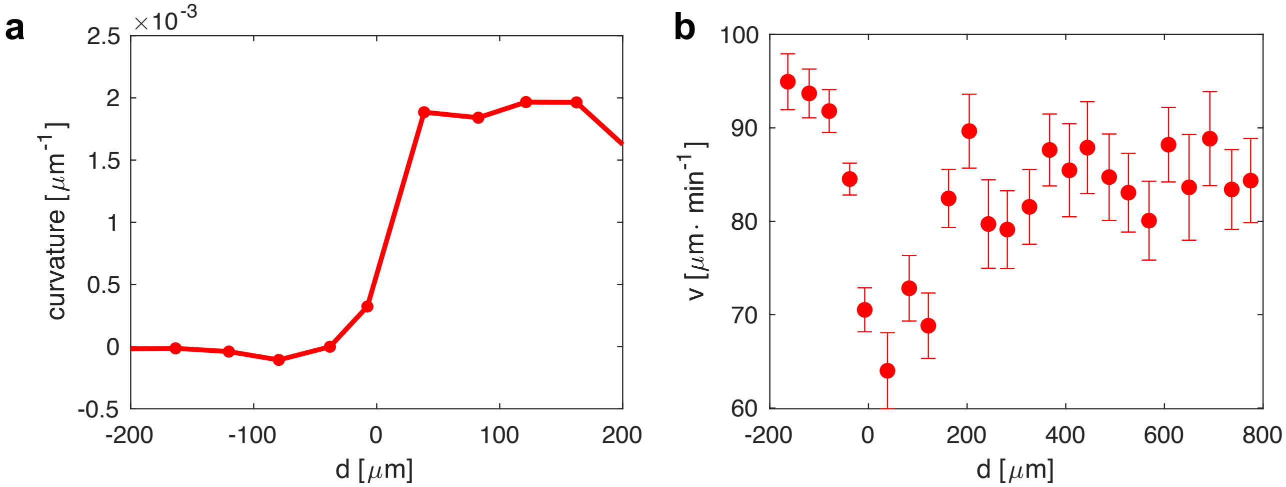

Fig. S1. Changes of curvature and velocity in transition to chiral gliding. a) The average curvature of all tracked trajectories as a function of the distance from the drop boundary. b) Velocity of the filaments as they cross the droplet interface. $d = 0$ represents the boundary of the droplet, negative and positive values of d represent the distance of the leading filament end inward or outward from the interface of the droplet.

In the main text, we have shown that the velocities of chiral gliding filaments are on average slower than those of straight filaments or those that glide back on their slime traces. However, we addressed the question how the velocities change in the course of the chiral gliding process. The velocity is lowest during the initiation of the bend. This is consistent with our proposed theoretical model for filament curvature.

Time-integrated filament density and circular motion patterns. Figure S2 illustrates the density plot accumulated over the entire simulation time, providing a time-integrated view of filament trajectories (shown in Fig. 5 (d-g) in the main text). It shows that the filaments increasingly follow circular paths, as evidenced by the pronounced ring-like structures in the 2D spatial density plot. These patterns indicate that the filaments are not randomly distributed but tend to orbit within confined radial zones. This spatial map highlights the emergence of organized, collective circular motion.

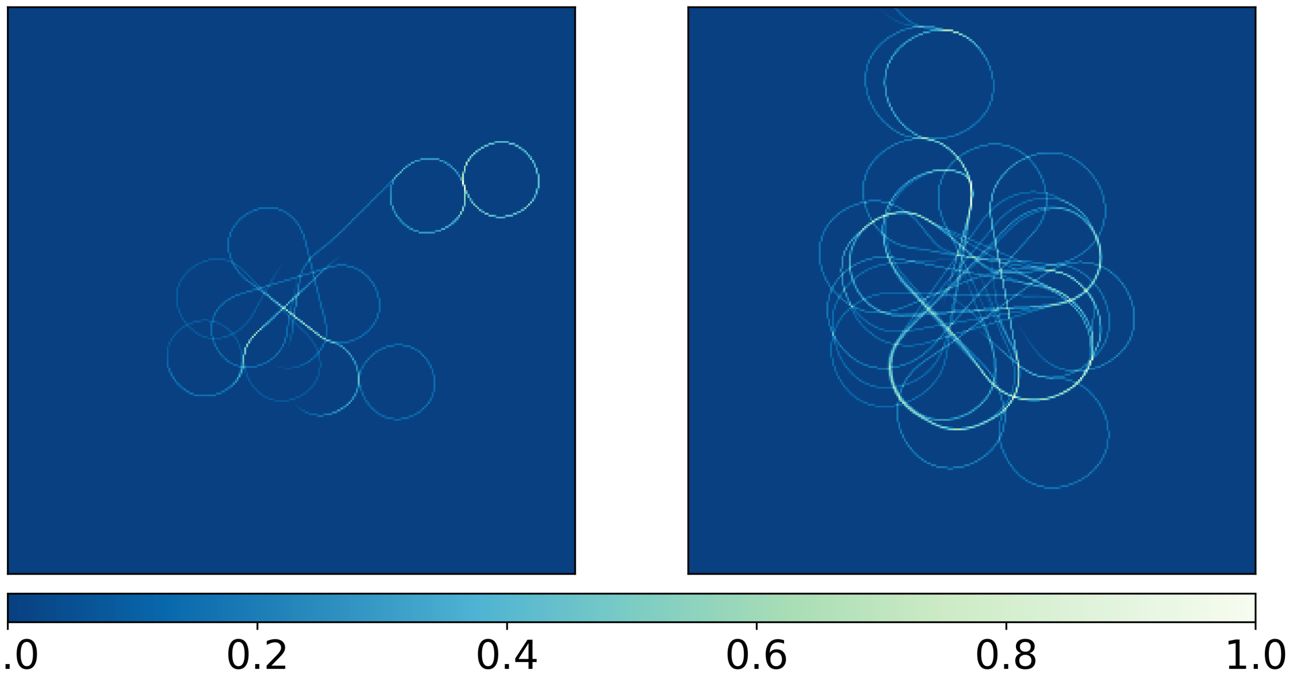

Fig. S2. Time-integrated 2D density plot of filament positions corresponding to Fig. 5. Brighter regions indicate higher occupancy, revealing the emergence of organized circular motion over the course of the simulation. The accompanying color bar represents the normalized positional density, with brighter regions indicating higher filament occupancy and longer residence times.

Chiral gliding guides the filaments back to the hydrated environment. We have introduced a dense population of filaments inside a droplet. In a dense population the filaments leave the droplet in the form of long bundles and keep their interaction with each other, resulting in a continuous stream of filaments emerging from the droplet. The bundles also show right-handed bending similar to the single filaments and the continuity of the bundle results in navigation of the filaments back to the droplet volume, a dynamics that continues over long time (Figure S3, Video S9). In addition, the filament bundles then show linear gliding inside the droplet. This suggests that the filaments can use the chiral gliding to navigate back to the droplet. In other words, the chiral gliding, with or without reversal, can then make the filaments avoid unfavorable conditions and steer back to the hydrated environment.

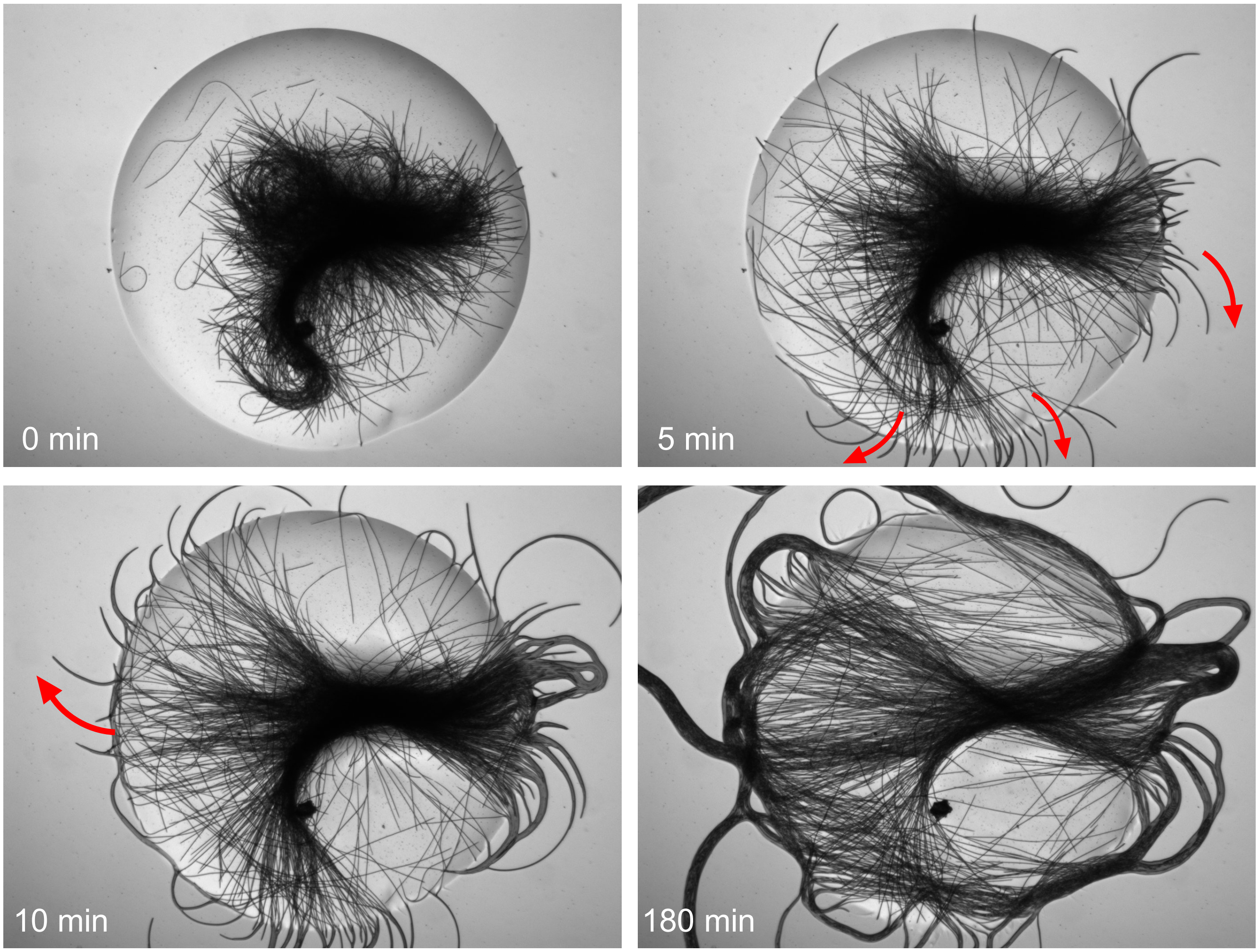

Fig. S3. Chiral gliding and recirculation of the filaments within the droplet in a densely populated droplet. The right-handed bending ensures that the filaments do not leave the main population within the droplet.

Right-handed chirality of the filaments along their long axis, reversal only changes the propagation end. Fig. S3 demonstrates how the chirality of the rotation of the bacteria is equivalent to a right-handed screw. From both ends of the screw, clockwise rotation pushes the screw forward through the nut. The same logic is also seen in the spring case, from two ends of the spring the spring wire continues in a clockwise rotation. This suggests that the pili grow and exert force on the filament at an angle angle that rotates by exactly 180 degrees when the filament reverses the gliding direction. In the alternative model, the reversal shifts the propagation of the slime from one end to another end, yet in the same angle.

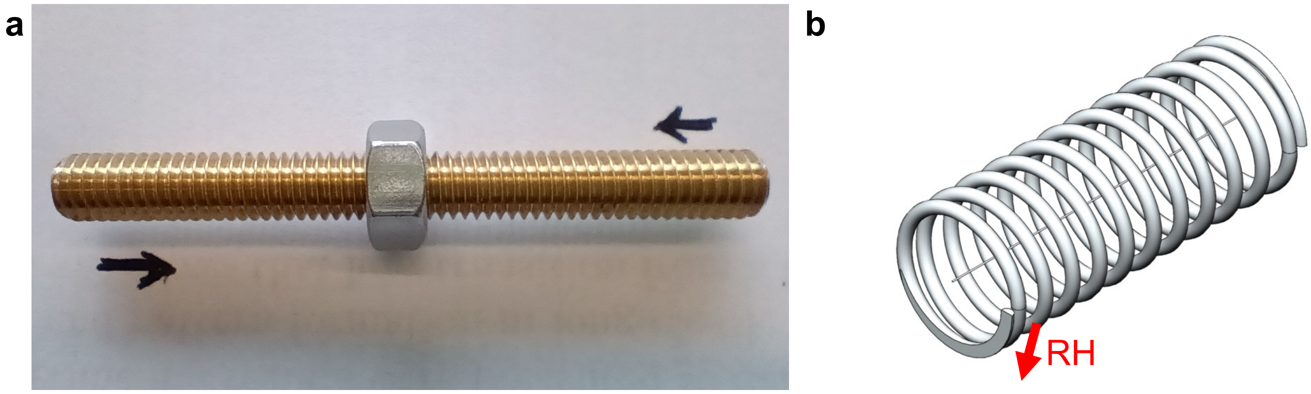

Fig. S4. Demonstration of the analogy between the right-handed chirality seen in cyanobacteria and a right-handed screw or spring right-handed chirality. a) shows a screw-nut and highlights that the clockwise rotation from either end push the screw forward. b) right-handed spring and the clockwise rotation of the spring wire from both ends.

Movie S1. Nonpolar gliding of a single filament on the bottom of a Petri dish

Movie S2. Right-handed turning of the filaments upon leaving the droplet

Movie S3. Reconstruction of the slime traces

Movie S4. Evaporating droplet experiment showing chiral gliding of the filaments

Movie S5. Buckling of the filaments at the interface of the droplet on soft agar

Movie S6. Clockwise rotation of the filament about its long axis

Movie S7. Simulation results of chiral gliding filaments (2 filaments)

Movie S8. Simulation results of chiral gliding filaments (6 filaments)

Movie S9. Chiral gliding and recirculation of filaments within the droplet in a densely populated droplet
